# Effects from containment and closure policies to market quality: Do they really matter in Vietnam during Covid-19?

**DOI:** 10.1371/journal.pone.0248703

**Published:** 2021-04-01

**Authors:** Duc Hong Vo, Bao Doan

**Affiliations:** 1 The CBER – Research Centre in Business, Economics & Resources, Ho Chi Minh City Open University, Ho Chi Minh City, Vietnam; 2 Department of Economics and Finance, Royal Melbourne Institute of Technology, Ho Chi Minh City, Vietnam; The Bucharest University of Economic Studies, ROMANIA

## Abstract

During the Covid-19 pandemic, the Vietnamese government has actively implemented various policies to achieve dual objectives: (i) to minimize the loss of life due to the infection; and (ii) to support economic growth. This paper is conducted to examine the effect of the government’s containment and closure policy on the stock market quality in Vietnam. Unlike other papers, we focus exclusively on market quality during the pandemic. We find that the policies appear to positively affect the market quality, except for closing-school policy (negative effect) and international travel (no effect). We argue that the government should sustain the policies until the wide availability of the vaccine to support the stock market quality in the near future.

## 1. Introduction

Vietnam has generally been considered one of the few countries to control the widespread of the Covid-19 pandemic successfully. The country recorded the first confirmed case in the early January 2020, leading to a complete lockdown in the nation over the first two weeks of April 2020. The second wave of the pandemic hit the country again three months later in July 2020 in Danang’s tourist-hub city in the central part of Vietnam. However, the government had partially locked down the affected provinces/cities only with the conservative policies supporting the economy. In addition, Vietnam has adopted a low-cost strategy. Vietnam’s well-known strategy includes a rapid introduction of containment measures and aggressive and cost-effective control measures.

This paper examines the effects of containment and closure policy implemented by the Vietnamese government during the January-September 2020 period. Various policies concerning the containment of the virus’s spread and the closure of schools and public spaces are considered. Also, we consider various indicators which are widely used as the proxies for market quality. Findings from this paper will play an important role in stipulating and implementing policies in the future to balance the benefits/costs arising from a health crisis and the economy. We consider the issue raised in this paper is worth investigating for policy implications; in particular, the pandemic has provided no ending sign soon. Like any other country, the Vietnamese government has to consider various policy options to ensure that the dual objectives can be achieved by the minimal loss of life due to the pandemic and the supports required to enhance the national economy to grow. Unlike other papers on Covid19-related topics, this paper focuses exclusively on the Vietnamese stock market’s market quality during the pandemic.

The structure of the paper is as follows. Following this introduction, section 2 discusses related literature. Data and research methodology are presented in section 3. The paper’s empirical findings are presented and discussed in section 4, followed by concluding remarks and policy implications in section 5 of the paper.

## 2. Related literature

Various studies on Covid19-related topics have been conducted and published in 2020. For a majority of these empirical studies, the focus is on the effects of Covid-19 pandemic to stock markets’ performance and/or volatility [1]. investigated the stock market response to COVID– 19 pandemics by applying the OLS, fix-effect and random-effect on a sample containing 64 countries during a period January 22, 2020, to April 17, 2020. During the period, the author found solid evidence for the stock market’s negative response to the increase in fatalities. Findings from the paper imply that the stock market return is relatively sensitive to the information of newly infected cases. Similarly, in another paper [2], posited that the government announcement in relation to the quarantine policies, lockdown, travelling restriction might affect the stock market’s volatility. Using the price indices of 80 countries over January 22, 2020, to April 17, 2020, empirical evidence implies the social distancing policy results in either negatively direct effect or positively indirect effect on the stock markets since containments to some extent hinder the functionality of the economy.

At the narrower scope [3], focused on the effects of the increase in confirmed cases/deaths to the stock market’s performance. Controlling for macroeconomic conditions, these authors utilized firms’ characteristics, which are collected from Bloomberg over the period from January 10 to March 16, 2020, as controlled variables. Remarkably, the study also pointed out that the beverages and air transportation industries are more vulnerable to the increase in the confirmed case than other industries during the outbreak of COVID– 19 in Wuhan. On the contrary, the return on pharmaceutical industry stocks positively reacts to the news of fatalities/infected case during the investigated period.

In the context of Vietnam [4], found the asymmetric and positive effects of government response on stock market returns. This paper uses the sample of all listed firms on Hanoi and Ho Chi Minh City stock exchanges. They concluded that the quarantine policies, containment and closure exerted upward pressure on the stock market returns. The results also indicated that the lockdown is necessary for preventing any further damage caused by the pandemic. Furthermore, their findings also imply that Vietnamese investors’ trust in the government efforts on fighting back the contagious infectious virus. Lastly, the financial sector of Vietnam is one of the most affected sectors during the outbreak of that deadly virus because it has been attacked by a dramatic surge in outstanding loans derived from the economic stagnation, which leads to the increase in the provision for the expected loss. [5] found that stimulus package that aims to reduce the economic repercussion fortified investor confidence and, as such, increased stock market returns. In detail, the government responses regarding these travel bans, lockdowns, and stimulus package exert upward pressure on the stock market returns of the G7 countries. With OLS estimator’s utilisation on the stock market returns from the G7 countries over the period July 1, 2019, to April 16 2020, findings from the study further imply the essential role of the government policies in fighting back the global economic stagnation.

Stressing on the spill-over effects of COVID-19 [6], concluded that the shock caused by the new virus outbreak on the stock market remains only for the short-term. The authors also found evidence that the depressive effects are only targeted to the global stock market but nearly nondirective to any typical stock market. The paper uses the Mann-Whitney tests on the price indices of countries whose stock markets are influential and badly hit by the economic stagnation. These countries include USA, China, Italy, Japan, Germany and Korea. Findings from the analyses showed that the impact of economic depression initially derived from Asia countries and quickly transmitted to other European and American markets since late Feb 2020.

Not only the stock markets but also the markets of fixed income instruments are affected by the global pandemic [7]. Argued that the economic turmoil likely lessens the effectiveness of many stimulus packages since it is relatively difficult to issue government bonds to smooth public consumption. Moreover, emerging markets are affected more severely than those in developed countries. The author measured the impact of COVID-19 on debt issues using various types of descriptive indicators such as mean, median and standard deviation. Focusing mainly on the bid/ask spread of 2,061 securities, issued by both IG and HY issuer from 74 emerging countries, the author showed that COVID-19 pandemic caused a credit and liquidity shock for the bond markets in late March 2020.

Besides the conditional reaction of the stock market over the new confirmed cases/deaths, the responses of government for preventing any unwanted effects caused by the spread of the virus are also noticeable [8, 9]. Presented real-time data collected by Oxford students, officers and alumni. The extents to which the government reacts to the COVID-19 pandemic are measured in four distinct dimensions: containment and closure, economic response, health system and miscellaneous. There are multiple qualitative and quantitative indicators collected publicly through news and announcement across nations to reflect each of these aspects in term of newly implemented policies. The authors consider that it is important to develop a comparable composite index of government reactions.

Our literature review above indicates that the effect from Covid-19 pandemic to market quality appears to have been largely ignored in current literature. In addition, Vietnam is considered as a successful country where the spread of Covid-19 is well under control with a low-cost approach. However, the effects of the important policies have not been widely discussed so that lessons can be learnt. As such, this study is warranted to be conducted to provide additional and fresh evidence on the effect of Covid-19 on the quality of the stock markets.

## 3. Data and research methodology

### 3.1 Data and variables

We collect the trade and quote intraday data of all stocks traded on the Ho Chi Minh Stock Exchange (HSX) from Thomson Reuters during the trading hours from 9:15 am to 11:30 am and 1:00 pm to 2:30 pm over the January-September 2020 period. We retain the positive bid/ask quotes and positive bid-ask spread as a valid quote, and we aggregate all trading volumes that take place at the same time and price into one. We use the [10] algorithm to infer the trade direction (buy or sell trades) and measure market quality with different proxies following [11]. The quoted spread is simply the difference between the bid and ask prices, and the effective bid-ask spread is defined for each trade as twice the absolute difference between the trade price and the mid-point of the contemporaneous bid and ask quotes. The realized spread is computed as twice the amount by which prices for customer buy orders exceed, or prices for customer sell orders fall short of, the estimated post-trade value of the asset. The midpoint of the quotes in effect 30 minutes after the trade, or the 14:30 midpoints for trades executed after 14:00, is used as a proxy for the post-trade value. The quotation size is simply the size of bid and ask quote recorded during a trading day. Alternatively, we measure the traded spread and its components, namely inventory plus adverse selection cost and order-processing cost, following [12]. Let *Q*_*t*_ be the buy-sell indicator at time *t* that equals one if the transaction is buyer-initiated and -1 if the transaction is seller-initiated, and *P*_*t*_ be the actual observed transaction price at time *t*. In a given day for each stock, the traded spread *S* and the percentage of *S* attributable to the adverse-selection and inventory cost *λ* in a day for a given stock are estimated from the ordinary least square regression as follows:

$$\Delta \;P_{t}=\lambda SQ_{t-1}+\frac{S}{2}(Q_{t}-Q_{t-1})+\epsilon_{t}$$ (1)

Given the estimates of *S* and *λ*, the order-processing cost is *S*(1 − *λ*). To aggregate the market-wide level of market quality measures, a simple mean is first computed on a stock daily basis, except for the quoted spread and quotation size that are time-weighted average. The cross-sectional daily mean is then calculated to have a time series observation, so this gives the results that pertain to an average trade in average sample stock.

The daily COVID-19 confirmed case data is obtained from the Coronavirus Resource Centre website of the John Hopkins University. From the Oxford COVID-19 Government Response Tracker (OxCGRT) database, we collect the individual policy responses that are related to containment and closure policies [8, 9]. The higher the index value, the stricter the policy, and the value of zero indicates no policy to be applied. In particular, the containment and closure policies include *school closing*, *workplace closing*, *cancel public events*, *restrictions on gatherings*, *close public transport*, *stay at home requirements*, *restrictions on the internal movement and international travel controls*. In addition, we obtain the *stringency index* that aggregates all individual policies above plus *public information campaigns*. We also collect the mobility trends reports consisting of indexes on driving and walking of users in Ha Noi and Ho Chi Minh city from Apple to proxy for the commuting activities. We sum all these individual indexes to form the aggregate mobility index or *mobility* of the country, whose higher level indicates more requests for directions in Apple Maps.

### 3.2 Methodology

**Fig 1:**
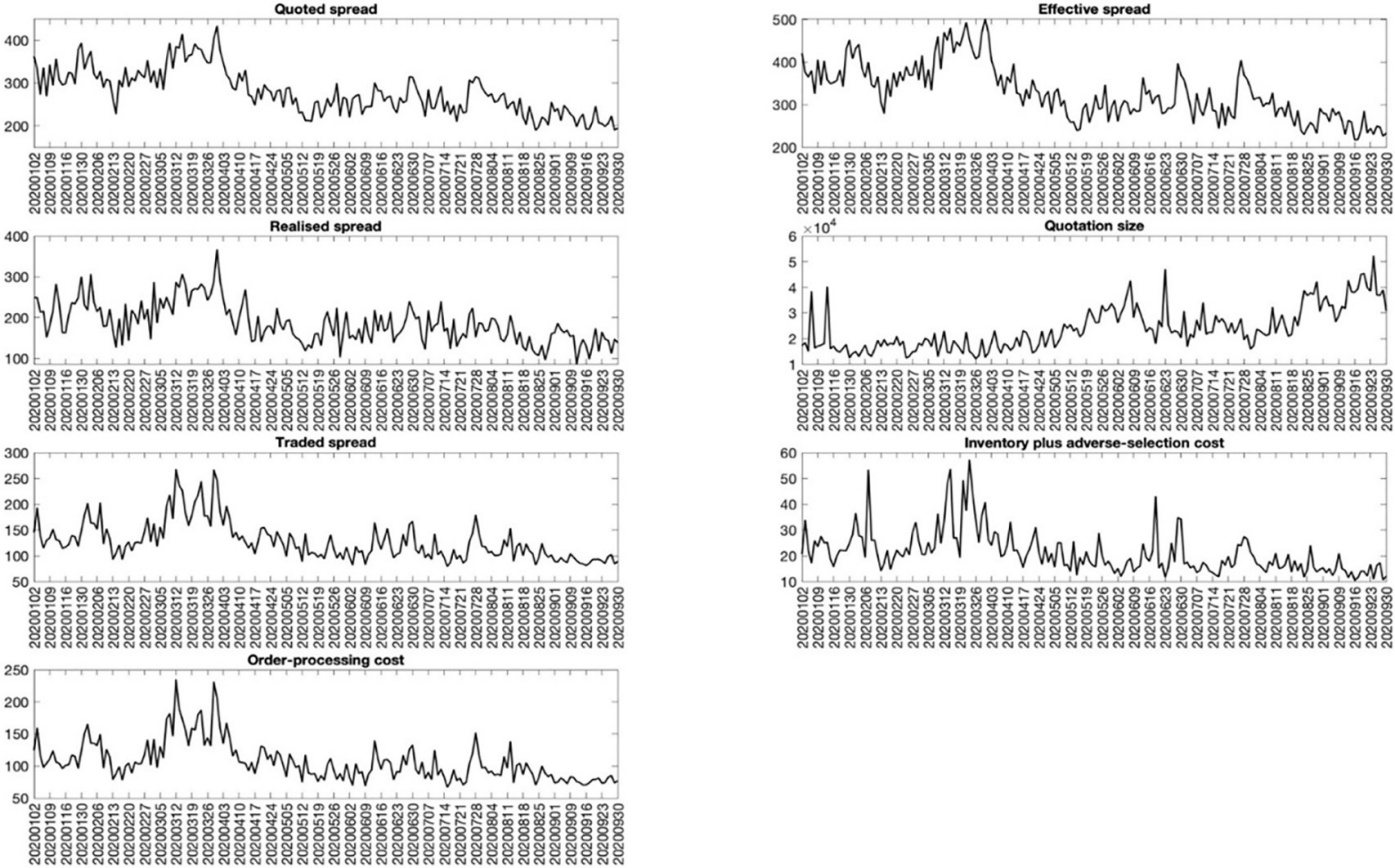
The market quality measures over January to September 2020. This figure presents different market quality measures daily over the period of January 2020 to September 2020. The intraday transaction data of all stocks listed in HSX is obtained to calculate the market quality measures. The quoted spread is simply the difference between the bid and ask prices, and the effective bid-ask spread is defined for each trade as twice the absolute difference between the trade price and the mid-point of the contemporaneous bid and ask quotes. The realized spread is computed as twice the amount by which prices for customer buy orders exceed, or prices for customer sell orders fall short of, the estimated post-trade value of the asset. The quotation size is simply the size of bid and ask quote recorded during a trading day. The traded spread and its components, namely inventory plus adverse selection cost and order-processing cost, are estimated following Huang and Stoll (1997). To aggregate the market-wide level of market quality measures, a simple mean is first computed on a stock daily basis, except for the quoted spread and quotation size that are time-weighted average. The cross-sectional daily mean is then calculated to have a time series observation.

**Fig 2:**
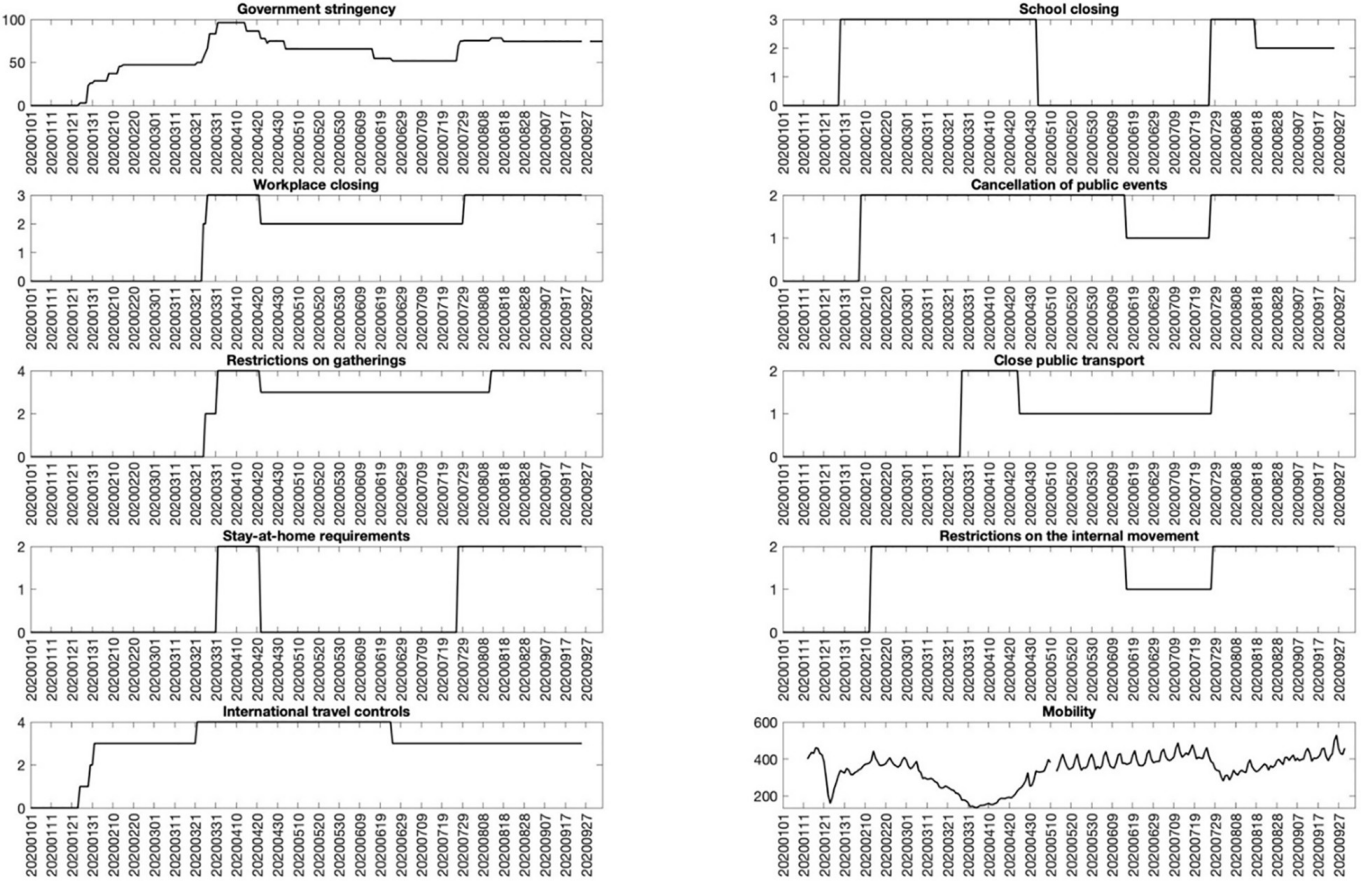
The government policy measures over January to September 2020. This figure presents different government policy measures on a daily basis over the period of January 2020 to September 2020. The stringency index and containment and closure policies are obtained from the Coronavirus Resource Centre website of the Oxford COVID-19 Government Response Tracker (OxCGRT) database. The Apple’s mobility indexes on driving and walking users in Ha Noi, Vietnam’s capital city and Ho Chi Minh city, the country’s economic and business centre, are summed up to be the aggregate mobility index.

For each measure of market quality, we run the following regression:

$$\begin{array}{l} {\text{Y}}_{\text{t}}=\beta_{0}+\beta_{1}Government\;Response_{t}+\beta_{2}\;Confirmed\;Case_{t-1}\\ \;+\beta_{3}Government\;Response_{t}\times Confirmed\;Case_{t-1}+\epsilon_{t}, \end{array}$$ (2)

where Y_t_ denotes a proxy of market quality on day *t*, *Government Response*_*t*_ represents the policy index on day *t*, and *Confirmed Case*_*t*_ is the number of newly confirmed cases on day *t*. The individual containment and closure policies in Fig 2 are similar to the ordinal variable whose higher-order indicates a stricter response of the government, so the coefficient *β*_1_ illustrates the additional impact of more rigorous policy. We note that the higher mobility index implies more demand for direction search in Apple Maps. Hence, the coefficient *β*_1_ suggests the additional effect of such a high number of requests for direction search (or equivalently fewer limitations on commute) on stock market quality [12]. documents that the daily number of newly confirmed cases matters the stock market returns, so it is likely that this variable also has an impact on stock market quality through the coefficient *β*_2_. In addition, we add the interaction term to study if the government policy helps remedy the impact of newly confirmed cases on the stock market. The day-of-the-week dummies are also included, and the robust standard errors are used to calculate t-statistics to control for volatility clustering in the proxies of market quality in Fig 1. To assess the economic impact of different policies, we divide all the daily observations of each variable by its standard deviation, so the regression estimates suggest how much change in terms of standard deviation in the dependent variable from a one-standard-deviation increase in the explanatory variable.

## 4. Results

### 4.1 Summary statistics

Table 1 presents the summary statistics of market quality measures and different government policies. The daily average quoted, effective and realized spreads are 187 to 328 Vietnam dong (VND) and the average quotation size is around 23,895 shares. The daily average traded spread, inventory plus adverse-selection cost, and the order-processing cost is 128, 21, and 107 VND, respectively, indicating that the order-processing cost contributes to the traded spread to a greater extent. All the market quality measures exhibit positive skewness, and the traded spread and its components have heavier tails than the other proxies of market quality. Also, the standard deviations of market quality measures are less than their average by 50 to 80%, suggesting that the variables’ fluctuations are not extreme. We observe a lower market quality, suggested by the higher spread-related variables and smaller quotation size, at the start of COVID-19 in February-March 2020 and the lockdown of Da Nang city in late July 2020 as shown in Fig 1. For example, when schools were first announced to stay shut nationwide on March 16, 2020, or the country started to suspend all inbound flights on March 21 2020, the quoted spread widens, and quotation size reduces to a much lower level over a short number of days. In contrast, when the government decided to ban all cultural, sports and entertainment activities at public places on March 28, 2020, and gathering of more than two people on April 1 2020, it is clear that the quoted spread becomes narrower after reaching its highest value at the end of March 2020.

**Table 1:** Summary statistics.

	Nobs	Mean	Median	Stdev	Skewness	Kurtosis	Min	Max
Quoted spread (Vietnam don VND)	186	279.15	271.40	52.87	0.50	2.68	189.53	433.33
Effective spread (VND)	186	327.91	319.61	64.10	0.56	2.67	217.35	501.41
Realized spread (VND)	186	186.74	178.71	49.57	0.63	3.20	85.75	367.33
Quotation size (shares)	186	23,895.01	22,222.41	8,372.85	0.88	3.17	12,186.33	52,385.83
Traded spread (VND)	186	127.86	118.10	37.55	1.39	5.04	79.53	267.85
Inventory plus adverse-selection cost (VND)	186	21.12	19.32	8.39	1.79	6.95	10.47	57.25
Order-processing cost (VND)	186	106.74	100.55	30.68	1.47	5.65	67.03	234.75
Stringency index	269	57.04	65.74	24.67	-0.96	3.50	0	96.30
School closing	269	1.61	2	1.40	-0.19	1.17	0	3
Workplace closing	269	1.69	2	1.21	-0.44	1.62	0	3
Cancellation of public events	269	1.57	2	0.73	-1.33	3.18	0	2
Restrictions on gatherings	269	2.28	3	1.61	-0.55	1.61	0	4
Close public transport	269	1.00	1	0.81	-0.01	1.54	0	2
Stay-at-home requirements	269	0.61	0	0.92	0.85	1.72	0	2
Restrictions on the internal movement	269	1.52	2	0.76	-1.20	2.81	0	2
International travel controls	269	3.04	3	1.11	-1.72	5.40	0	4
Mobility index	260	344.24	363.85	84.87	-0.86	3.03	133.96	528.50
# confirmed cases	256	4.28	1	8.37	3.01	13.15	0	50

This table presents the summary statistics of different variables daily over the period of January 2020 to September 2020. The intraday transaction data of all stocks listed in HSX is obtained to calculate the market quality measures. The quoted spread is simply the difference between the bid and ask prices, and the effective bid-ask spread is defined for each trade as twice the absolute difference between the trade price and the mid-point of the contemporaneous bid and ask quotes. The realized spread is computed as twice the amount by which prices for customer buy orders exceed, or prices for customer sell orders fall short of, the estimated post-trade value of the asset. The quotation size is simply the size of bid and ask quote recorded during a trading day. The traded spread and its components, namely inventory plus adverse selection cost and order-processing cost, are estimated following Huang and Stoll (1997). To aggregate the market-wide level of market quality measures, a simple mean is first computed on a stock daily basis, except for the quoted spread and quotation size that are time-weighted average. The cross-sectional daily mean is then calculated to have a time series observation. The daily number of a newly confirmed case, stringency index, and containment and closure policies are obtained from the Coronavirus Resource Centre website of the John Hopkins University and the Oxford COVID-19 Government Response Tracker (OxCGRT) database, respectively. The Apple’s mobility indexes on driving and walking of users in Ha Noi, Vietnam’s capital city and Ho Chi Minh city, the country’s economic and business centre, are summed up to be the aggregate mobility index.

Regarding the individual containment and closure policies, these variables, except for *stay-at-home requirements* and *close public transport*, exhibit negative skewness. These findings imply that a majority of daily observations are located on the right tail, or the government policies are implemented to a great extent in our sample period. The *stringency index*, *cancellation of public events* and *international travel controls* demonstrate excessive kurtosis, indicating that these variables are more likely to yield extreme observations. Interestingly, the negative skewness in the mobility index suggests a high daily demand for direction search in our sample period, where its median of 363.85 is higher than its average of 344.24. Lastly, the number of newly confirmed cases is average, 4 cases per day, and exhibits more extreme cases by excessively high kurtosis.

Fig 2 illustrates the time series behaviours of different containment and closure policies to show that these variables are closely related to each other. Four patterns are noticed from the correlation between the market quality proxies and government policies in Table 2. First, the government responses, except for *school closing*, are positively correlated with market quality measures, implied by the negative correlation with spread-related variables and positive correlation with quotation size. In contrast, the stricter school closing indicates lower market quality via the positive (negative) correlation with the spread (quotation size). Second, the stringency index by construction is highly correlated with the individual policies whose correlations are above 0.6 except with *school closing*. Third, *workplace closing*, *restrictions on gatherings*, *close public transports*, and *stay-at-home requirements* are highly correlated with each other with a correlation above 0.5, indicating that these policies are likely applied at the same time. Not surprisingly, *cancellation of public events*, *restrictions on the internal movement*, and *international travel controls* also have their correlations above 0.5, and these policies belong the rule set of social distance. Last but not least, the mobility index is negatively correlated with all government responses, suggesting that Apple users tend to search more for directions and hence are likely to commute when the policies are less restrictive.

**Table 2:** The correlation matrix.

	Stringency index	School closing	Workplace closing	Cancellation of public events	Restrictions on gatherings	Close public transport	Stay-at-home requirements	Restrictions on the internal movement	International travel controls	Mobility index
Quoted spread	-0.35	0.38	-0.58	-0.18	-0.67	-0.54	-0.37	-0.19	-0.14	-0.48
Effective spread	-0.33	0.40	-0.57	-0.17	-0.66	-0.53	-0.35	-0.19	-0.12	-0.49
Realized spread	-0.30	0.27	-0.45	-0.21	-0.54	-0.46	-0.33	-0.21	-0.11	-0.41
Quotation size	0.29	-0.34	0.50	0.19	0.56	0.47	0.36	0.22	0.08	0.44
Traded spread	-0.15	0.41	-0.40	-0.03	-0.49	-0.36	-0.26	-0.05	0.05	-0.53
Inventory plus adverse-selection cost	-0.19	0.32	-0.40	-0.07	-0.47	-0.38	-0.27	-0.09	0.05	-0.44
Order-processing cost	-0.13	0.41	-0.38	-0.02	-0.46	-0.33	-0.24	-0.03	0.05	-0.53
Stringency index		0.26	0.82	0.74	0.79	0.82	0.65	0.75	0.72	-0.43
School closing			-0.10	0.39	-0.24	0.06	0.42	0.31	0.06	-0.53
Workplace closing				0.37	0.96	0.94	0.70	0.43	0.41	-0.13
Cancellation of public events					0.32	0.39	0.38	0.91	0.61	-0.30
Restrictions on gatherings						0.91	0.63	0.38	0.43	-0.03
Close public transport							0.81	0.44	0.31	-0.16
Stay-at-home requirements								0.38	0.02	-0.19
Restrictions on the internal movement									0.59	-0.31
International travel controls										-0.41

This table presents the correlation matrix of different variables on a daily basis over the period of January 2020 to September 2020. The intraday transaction data of all stocks listed in HSX is obtained to calculate the market quality measures. The quoted spread is simply the difference between the bid and ask prices, and the effective bid-ask spread is defined for each trade as twice the absolute difference between the trade price and the mid-point of the contemporaneous bid and ask quotes. The realized spread is computed as twice the amount by which prices for customer buy orders exceed, or prices for customer sell orders fall short of, the estimated post-trade value of the asset. The quotation size is simply the size of bid and ask quote recorded during a trading day. The traded spread and its components, namely inventory plus adverse selection cost and order-processing cost, are estimated following Huang and Stoll (1997). To aggregate the market-wide level of market quality measures, a simple mean is first computed on a stock daily basis, except for the quoted spread and quotation size that are time-weighted average. The cross-sectional daily mean is then calculated to have a time series observation. The stringency index and containment and closure policies are obtained from the Coronavirus Resource Centre website of the Oxford COVID-19 Government Response Tracker (OxCGRT) database, respectively. The Apple’s mobility indexes on driving and walking of users in Ha Noi, Vietnam’s capital city and Ho Chi Minh city, the country’s economic and business centre, are summed up to be the aggregate mobility index.

### 4.2 Main results

Table 3 reports the results with different measures of government responses. The correlation table in Table 2 presents the individual policies into three main groups according to their correlation magnitude and interprets the results accordingly. These groups are *school closing* (1); *workplace closing*, *restrictions on gatherings*, *close public transport*, and *stay-at-home requirements* (2); and *cancellation of public events*, *restrictions on the internal movement*, and *international travel controls* (3). In regard to policy group (1), it exerts a negative impact on market quality in that a standard deviation increase in *school closing* is associated with an increase of 0.35 to 0.5 standard deviation in the spread-related variables and a decrease of 0.41 standard deviation in the quotation size at 1% significance level. Moving to the policy group (2), the impact is now reverse in that the stricter policy enhances the market quality, and the results are significant at 1% level. In particular, a standard deviation increases in *workplace closing* or *restrictions on gatherings* is associated with a decrease of 0.64 to 0.66 standard deviation in the quoted and effective spreads, a reduction of 0.43 to 0.47 standard deviation in the realized and traded spreads and its components, and an increase of 0.66 standard deviations in the quotation size. The degree of impact on market quality decreases slightly with the control of *close public transport* but strongly with *stay-at-home requirements*. For example, a standard deviation increase in *stay-at-home requirements* reduces 0.28 to 0.35 standard deviation in the quoted, effective and realized spreads, 0.19 to 0.23 standard deviation in the traded spread and its component, but increases 0.45 standard deviation in the quotation size. With respect to the last policy group (3), these government responses yield a positive impact on market quality to a less extent, given the statistically significant results at 5% to 10% level. A standard deviation increase in *cancellation of public events* or *restrictions on the internal movement* is approximately associated with a reduction of 0.24 to 0.29 standard deviation in the quoted, effective and realized spreads. It is also associated with a decrease of 0.19 to 0.22 standard deviation in the traded spread and its components, and an increase of 0.29 standard deviation in the quotation size. For *international travel control*, a standard deviation increase reduces 0.41 to 0.45 standard deviation in the quoted, effective and realized spreads at the 5% significance level. With respect to *mobility index*, its impact on market quality is stronger than that of policy group (3) and statistically significant at 1% level. In particular, a standard deviation increase in *mobility* decreases 0.43 to 0.52 standard deviation in the quoted, effective and traded spreads and its components, a reduction of 0.24 standard deviation in the realized spread and an increase of 0.57 standard deviation in the quotation size.

**Table 3:** The impact of different containment and closure policies on market quality.

	Government response	t-stat	Lagged confirmed case	t-stat	Interaction term	t-stat	Intercept	t-stat
School closing								
Quoted spread	0.497	[7.71]***	-0.194	[-1.47]	0.073	[1.09]	5.038	[30.95]***
Effective spread	0.510	[7.86]***	-0.195	[-1.52]	0.069	[1.04]	4.890	[28.84]***
Realised spread	0.349	[4.72]***	-0.216	[-1.52]	0.091	[1.24]	3.468	[19.01]***
Quotation size	-0.410	[-6.44]***	-0.098	[-0.83]	0.058	[0.99]	3.450	[19.04]***
Traded spread	0.475	[6.33]***	-0.178	[-1.65]	0.070	[1.11]	3.213	[16.83]***
Inventory plus adverse-selection cost	0.412	[4.86]***	-0.157	[-1.37]	0.030	[0.49]	2.380	[11.65]***
Order-processing cost	0.468	[6.32]***	-0.175	[-1.58]	0.077	[1.19]	3.273	[17.3]***
Workplace closing								
Quoted spread	-0.661	[-8.7]***	0.586	[2.84]***	-0.151	[-1.76]*	6.522	[33.96]***
Effective spread	-0.636	[-8.45]***	0.740	[3.47]***	-0.224	[-2.56]**	6.337	[32.85]***
Realised spread	-0.474	[-5.31]***	0.627	[2.91]***	-0.201	[-2.19]**	4.503	[20.41]***
Quotation size	0.663	[9.76]***	0.178	[1.67]*	-0.192	[-3.76]***	1.988	[13.9]***
Traded spread	-0.446	[-4.83]***	1.062	[3.85]***	-0.382	[-3.29]***	4.311	[17.99]***
Inventory plus adverse-selection cost	-0.440	[-4.66]***	0.901	[2.32]**	-0.344	[-2.15]**	3.405	[13.35]***
Order-processing cost	-0.424	[-4.63]***	1.051	[3.87]***	-0.372	[-3.24]***	4.335	[18.29]***
Cancellation of public events								
Quoted spread	-0.242	[-2.07]**	-0.951	[-2.52]**	0.391	[2.7]***	6.193	[18.2]***
Effective spread	-0.264	[-2.29]**	-1.042	[-2.79]***	0.424	[2.95]***	6.111	[17.74]***
Realised spread	-0.292	[-2.61]**	-0.501	[-1.12]	0.217	[1.28]	4.572	[13.85]***
Quotation size	0.283	[3.02]***	0.672	[2.69]***	-0.296	[-3.12]***	2.299	[9.29]***
Traded spread	-0.206	[-1.76]*	-1.320	[-4.34]***	0.528	[4.25]***	4.254	[11.82]***
Inventory plus adverse-selection cost	-0.224	[-1.57]	-0.986	[-2.57]**	0.378	[2.56]**	3.390	[8.36]***
Order-processing cost	-0.191	[-1.71]*	-1.343	[-4.29]***	0.541	[4.24]***	4.270	[12.21]***
Restrictions on gatherings								
Quoted spread	-0.652	[-8.95]***	0.751	[3.89]***	-0.331	[-3.35]***	6.541	[36.09]***
Effective spread	-0.628	[-8.76]***	0.850	[4.92]***	-0.389	[-4.35]***	6.360	[35.05]***
Realised spread	-0.461	[-5.24]***	0.857	[3.7]***	-0.413	[-3.47]***	4.494	[20.61]***
Quotation size	0.670	[10.56]***	0.137	[1.13]	-0.160	[-2.35]**	1.929	[13.48]***
Traded spread	-0.449	[-4.87]***	1.100	[4.08]***	-0.529	[-3.83]***	4.343	[18.45]***
Inventory plus adverse-selection cost	-0.438	[-4.58]***	0.977	[2.85]***	-0.502	[-2.89]***	3.425	[13.19]***
Order-processing cost	-0.429	[-4.69]***	1.078	[3.84]***	-0.510	[-3.52]***	4.368	[18.86]***
Close public transport								
Quoted spread	-0.604	[-7.49]***	0.466	[2.13]**	-0.098	[-1.08]	6.342	[32.88]***
Effective spread	-0.577	[-7.16]***	0.586	[2.43]**	-0.158	[-1.6]	6.163	[31.52]***
Realised spread	-0.460	[-5.3]***	0.562	[2.59]**	-0.171	[-1.89]*	4.406	[21.15]***
Quotation size	0.610	[7.32]***	0.046	[0.42]	-0.139	[-2.6]**	2.190	[14.47]***
Traded spread	-0.384	[-4.12]***	0.774	[2.95]***	-0.262	[-2.39]**	4.181	[18.33]***
Inventory plus adverse-selection cost	-0.391	[-4.2]***	0.713	[2.2]**	-0.266	[–2]**	3.282	[13.42]***
Order-processing cost	-0.362	[-3.91]***	0.751	[2.85]***	-0.248	[-2.24]**	4.210	[18.74]***
Stay-at-home requirements								
Quoted spread	-0.351	[-4.47]***	0.499	[1.79]*	-0.162	[-1.24]	5.778	[30.74]***
Effective spread	-0.317	[-3.95]***	0.533	[1.84]*	-0.191	[-1.4]	5.618	[29.29]***
Realised spread	-0.276	[-3.55]***	0.540	[1.93]*	-0.214	[-1.63]	3.981	[21.94]***
Quotation size	0.447	[4.77]***	-0.143	[-1.35]	-0.062	[-1.1]	2.702	[17.27]***
Traded spread	-0.209	[-2.74]***	0.667	[2.16]**	-0.282	[-1.96]*	3.815	[19.71]***
Inventory plus adverse-selection cost	-0.229	[-3.15]***	0.518	[1.93]*	-0.235	[-1.88]*	2.931	[13.7]***
Order-processing cost	-0.193	[-2.52]**	0.674	[2.14]**	-0.280	[-1.91]*	3.858	[20.42]***
Restrictions on the internal movement								
Quoted spread	-0.258	[-2.55]**	-0.913	[-2.47]**	0.393	[2.67]***	6.182	[21.86]***
Effective spread	-0.279	[-2.78]***	-0.960	[-2.58]**	0.410	[2.76]***	6.093	[20.95]***
Realised spread	-0.270	[-2.61]**	-0.398	[-0.88]	0.187	[1.06]	4.464	[15.44]***
Quotation size	0.334	[4.01]***	0.758	[3.22]***	-0.344	[-3.72]***	2.245	[10.72]***
Traded spread	-0.207	[–2]**	-1.254	[-4.13]***	0.523	[4.08]***	4.220	[13.65]***
Inventory plus adverse-selection cost	-0.231	[-1.89]*	-0.970	[-2.56]**	0.388	[2.55]**	3.364	[10.14]***
Order-processing cost	-0.190	[-1.89]*	-1.267	[-4.05]***	0.533	[4.04]***	4.235	[13.83]***
International travel controls								
Quoted spread	-0.407	[-2.19]**	-2.917	[-2.68]***	1.057	[2.64]***	6.791	[11.01]***
Effective spread	-0.452	[-2.48]**	-3.113	[-2.78]***	1.124	[2.72]***	6.791	[11.16]***
Realised spread	-0.412	[-2.2]**	-2.986	[-2.68]***	1.073	[2.61]**	5.062	[8.29]***
Quotation size	0.107	[0.57]	1.368	[2.8]***	-0.517	[-2.99]***	2.688	[4.3]***
Traded spread	-0.243	[-1.4]	-3.029	[-2.83]***	1.102	[2.79]***	4.429	[7.49]***
Inventory plus adverse-selection cost	-0.202	[-1.18]	-2.809	[-2.98]***	0.999	[2.87]***	3.402	[5.61]***
Order-processing cost	-0.242	[-1.38]	-2.933	[-2.71]***	1.074	[2.69]***	4.480	[7.63]***
Mobility index								
Quoted spread	-0.421	[-4.56]***	1.584	[3.88]***	-0.421	[-4.19]***	7.238	[17.01]***
Effective spread	-0.426	[-4.62]***	1.597	[3.71]***	-0.426	[-4.02]***	7.115	[16.82]***
Realised spread	-0.241	[-2.51]**	2.013	[4.26]***	-0.531	[-4.45]***	4.739	[11.23]***
Quotation size	0.572	[7.08]***	0.152	[0.58]	-0.037	[-0.54]	0.728	[2.08]**
Traded spread	-0.519	[-4.74]***	1.166	[1.96]*	-0.312	[-2.15]**	5.783	[11.46]***
Inventory plus adverse-selection cost	-0.478	[-4.33]***	0.731	[1.32]	-0.215	[-1.59]	4.741	[9.03]***
Order-processing cost	-0.504	[-4.61]***	1.223	[2.03]**	-0.322	[-2.18]**	5.769	[11.5]***
Stringency index								
Quoted spread	-0.617	[-4.41]***	-0.028	[-0.03]	0.069	[0.23]	7.175	[18.33]***
Effective spread	-0.593	[-4.21]***	0.224	[0.23]	-0.018	[-0.06]	6.973	[17.73]***
Realized spread	-0.503	[-3.59]***	0.197	[0.24]	-0.021	[-0.08]	5.138	[12.85]***
Quotation size	0.627	[4.55]***	0.847	[2.11]**	-0.349	[-2.53]**	1.371	[3.97]***
Traded spread	-0.351	[-2.42]**	0.710	[0.71]	-0.192	[-0.57]	4.632	[11.04]***
Inventory plus adverse-selection cost	-0.385	[-2.61]**	0.925	[0.95]	-0.286	[-0.89]	3.804	[8.58]***
Order-processing cost	-0.324	[-2.24]**	0.617	[0.61]	-0.157	[-0.46]	4.618	[11.17]***

This table presents coefficient estimates and t-statistics in square brackets of the following regression Y_t_ = *β*_0_ + *β*_1_*Government Response*_*t*_ + *β*_2_ *Confirmed Case*_*t*−1_ + *β*_3_*Government Response*_*t*_ × *Confirmed Case*_*t*−1_ + *ϵ*_*t*_. The intraday transaction data of all stocks listed in HSX is obtained over January to September 2020 to calculate the market quality measures. The quoted spread is simply the difference between the bid and ask prices, and the effective bid-ask spread is defined for each trade as twice the absolute difference between the trade price and the mid-point of the contemporaneous bid and ask quotes. The realized spread is computed as twice the amount by which prices for customer buy orders exceed, or prices for customer sell orders fall short of, the estimated post-trade value of the asset. The quotation size is simply the size of bid and ask quote recorded during a trading day. The traded spread and its components, namely inventory plus adverse selection cost and order-processing cost, are estimated following Huang and Stoll (1997). To aggregate the market-wide level of market quality measures, a simple mean is first computed on a stock daily basis, except for the quoted spread and quotation size that are time-weighted average. The cross-sectional daily mean is then calculated to have a time series observation. The daily number of a newly confirmed case, stringency index, and containment and closure policies are obtained from the Coronavirus Resource Centre website of the John Hopkins University and the Oxford COVID-19 Government Response Tracker (OxCGRT) database, respectively. The Apple’s mobility indexes on driving and walking of users in Ha Noi, Vietnam’s capital city and Ho Chi Minh city, the country’s economic and business centre, are summed up to be the aggregate mobility index. Standard errors are adjusted for heteroskedasticity, and the regression also includes the day-of-the-week dummies.

***, **, and * represent the statistical significance at 1 per cent, 5 per cent, and 10 per cent levels, respectively.

The lagged daily number of newly confirmed cases has an ambiguous impact on the market quality proxies when controlled for different policies. While it doesn’t have any statistically significant effect on market quality with *school closing* or policy group (1), its coefficient turns statistically significant in policy group (2). When controlled for *workplace closing* or *restrictions on gatherings*, a standard deviation increase is approximately associated with a surge of 0.59 to 0.74 standard deviation in the quoted and effective spreads. It is also associated with a rise of 0.63 to 1.06 standard deviation in the realized and traded spreads as well as its components at 1% significance level. However, the impact on market quality reduces in size and statistical with the controls of *close public transport* and *stay-at-home requirements*. In particular, a standard deviation increase of a number of newly confirmed cases is associated with a rise of 0.47 to 0.59 standard deviation in the quoted, effective and realized spreads and a surge of 0.52 to 0.77 standard deviation in traded spread and its components at the 5% to 10% significance level. In regard to the policy group (3), the negative impact on market quality documented above is surprisingly reverse. When controlled for *cancellation of public events* or *restrictions on the internal movement*, a standard deviation increase in the number of newly confirmed cases approximately reduces the quoted, effective and traded spreads and its components by 0.96 to 1.3 standard deviation, and increases the quotation size by 0.67 standard deviations at the 1% to 5% significance level. Such a positive impact on market quality is enhanced by 2 to 3 times in coefficient magnitude with *international travel controls*’ control. Lastly, the number of newly confirmed cases exerts a negative impact on market quality when controlled for *mobility*. A standard deviation increase of this variable is associated with a 1.22 to 2 standard deviation increase in the spread-related variables at the 1% to 5% significance level.

To further evaluate how government responses help alleviate the impacts on market quality of a number of newly confirmed cases, Table 3 presents the coefficient estimates of the interaction term. It is expected that the coefficient *β*_3_ is not statistically significant when controlled for *school closing* since the standalone coefficient *β*_2_ is also statistically insignificant. Regarding the policy group (2), the impact of the number of newly confirmed cases on the spread-related variables reduces by 25% to 38% (with the control of *workplace closing*) or 44% to 52% (with the control of *restrictions on gatherings*) at times of stricter government responses at the 1% to 5% significance level. The policies of *close public transport* and *stay-at-home requirements* weakly reduce the negative impact of a number of newly confirmed cases in that the coefficient *β*_3_ only remains statistically significant at 5% to 10% level. Concerning the policy group (3), the interaction term also carries the sign opposite to standalone term of a number of a new confirmed case. In particular, the coefficient of the interaction term is 36% to 50% the magnitude of standalone term at the 1% significance level. Lastly, the negative effect of the number of newly confirmed cases on the spread-related variables and order-processing costs reduce by 26% at times of higher demand for direction search in Apple Maps.

The results in Table 3 lead to three different conclusions. First, market quality is not always enhanced by stricter containment and closure policies. In particular, *school closing* has a negative impact on market quality, where a standard deviation increase in this policy is associated with an increase of 0.35 to 0.5 standard deviation of spread-related variables and a decrease of 0.41 standard deviation of quotation size. Among the other policy measures, *workplace closing* and *restrictions on gatherings* exert the strongest positive effect on market quality, where a standard deviation increase of each response is associated with a decrease of 0.43 to 0.66 standard deviation of spread-related variables and an increase of 0.66 standard deviations of quotation size. This suggests that the market does not highly appreciate *school closing* since the parents have to take care of their children, potentially reducing economic activities and productivity. In addition, in the presence of policies on *workplace closing*, *restrictions on gatherings*, *close public transport*, *stay-at-home requirements*, and *restrictions on the internal movement*, both informed and uninformed traders are more willing to trade, leading to the reduction of inventory plus adverse-selection cost and order-processing cost. Second, the higher demand for search direction, an indirect measure of the ability to commute, helps enhance market quality since it potentially signals that the economic activities go back to normal operation. Lastly, the presence of *workplace closing*, *restrictions on gatherings*, *close public transport*, and less limitation on the commute (via higher demand on direction search) alleviate the negative impact of a number of confirmed cases on market quality.

### 4.3 Robustness

Table 3 documents that the individual policies exert different impacts on market quality, so it is interesting to see the net effects when multiple government responses are studied together. We answer this question with two different approaches. We first replace the individual policies in Eq (2) by the *stringency* index that aggregates all the containment and closure policies plus *public information campaign*. The results on the last panel of Table 3 documents that a stricter government response in containment and closure policies helps improve the market quality. A standard deviation increase in *stringency* reduces the quoted, effective and realized spread by 0.5 to 0.62 standard deviation, the traded spread and its components by 0.32 to 0.38 standard deviation, but increases the quotation size by 0.63 standard deviations at the 1% to 5% significance level. The results are in line with Table 3 because the policy group (2) that yields the positive impact on market quality have a greater contribution to the construction of *stringency* index in terms of a number of policies. More interestingly, the lagged number of newly confirmed cases and their interaction with *stringency* no longer has a statistically significant impact on market quality proxies. An exception is that a standard deviation increase in the standalone term surprisingly improves the quotation size by 0.85 standard deviations, but the effect is alleviated by 41% at times of stricter *stringency* at the 5% significance level.

We alternatively include all individual policies in the same regression and run the following model:

$${\text{Y}}_{\text{t}}=\beta_{0}+\Sigma_{i=1}^{8}\beta_{i}c_{i,t}+\gamma \;Confirmed\;Case_{t-1}+\epsilon_{t},$$ (3)

where Y_t_ denotes a proxy of market quality on day *t*, *c*_*i*,*t*_ represents the government response *i* on day *t*, and *Confirmed Case*_*t*_ is the number of newly confirmed cases on day *t*. We exclude the interaction terms for two reasons. First, Table 3 reports the mixing signs of the standalone and interaction terms associated with the number of newly confirmed cases across different individual policies. Second, given that many policies are effective at the same time, it is unclear on the interpretation of interaction term if changing one policy and fixing the others at a time. We overcome this by studying the interaction term between a number of newly confirmed cases and *stringency* documented above.

The results in Table 4 illustrates several main findings. First, the lagged number of a new confirmed case does not yield any statistically significant impact on the proxies of market quality, except that its one standard deviation increase is associated with a reduction of 0.2 standard deviations in quotation size. Second, the *school closing* policy continues to exert a negative impact on market quality where its one standard deviation increase is associated with a rise of 0.48 to 0.56 standard deviation in spread-related variables and order-processing cost. It is also associated with a surge of 0.29 standard deviation in inventory plus adverse-selection cost and a reduction of 0.76 standard deviations in quotation size. The results are statistically significant at the 1% level, except for the inventory plus adverse-selection cost significant at the 10% level. Third, only *restrictions on gatherings* of the policy group (2) remain its positive impact on market quality with a larger coefficient magnitude than that of *school policy*. A standard deviation increase of *restrictions on gatherings* reduces the quoted, realized and traded spreads and its components by 0.78 to 1.1 standard deviation at the 1% to 10% significance level. Also, the stricter *stay-at-home requirements* help improve the quotation size at the 5% significance level, and the other two policies of *workplace closing* and *close public transports* turn statistically insignificant in most cases. Fourth, only *cancellation of public events* in policy group (3) enhances market quality as its one standard deviation increase is associated with 0.45 to 0.49 standard deviation reduction in the spread-related variables and an increase of 0.25 standard deviation in the quotation size at the 1% to 5% significance level. This coefficient magnitude generally is of similar size of that from *school closing*, except for quotation size. While *restrictions on the internal movement* do not yield any statistically significant impact on market quality, *international travel controls* surprisingly depresses market quality to a greater extent than *school closing*. In particular, its one standard deviation increase is associated with an increase of 0.48 to 0.97 standard deviation in the spread-related variables and a reduction of 0.52 standard deviation in the quotation size at the 1% significance level in most cases.

**Table 4:** The impact of different containment and closure policies on market quality in the multivariate regression.

	Quoted spread	Effective spread	Realized spread	Quotation size	Traded spread	Inventory plus adverse-selection cost	Order-processing cost
School closing (c1)	0.533	0.539	0.479	-0.760	0.527	0.289	0.564
	[4.12]***	[3.85]***	[3.51]***	[-6.23]***	[3.89]***	[1.98]*	[4.02]***
Workplace closing (c2)	0.279	0.193	0.684	0.144	0.122	0.286	0.072
	[1.1]	[0.77]	[2.26]**	[0.9]	[0.38]	[0.87]	[0.19]
Cancellation of public events (c3)	-0.453	-0.478	-0.496	0.246	-0.470	-0.468	-0.447
	[-3.81]***	[-3.29]***	[-3.01]***	[2.19]**	[-3.44]***	[-2.64]***	[-3.12]***
Restrictions on gatherings (c4)	-0.801	-0.688	-0.778	-0.242	-0.818	-1.106	-0.699
	[-2.06]**	[-1.64]	[-1.68]*	[-0.87]	[-1.72]*	[-2.93]***	[-1.3]
Close public transport (c5)	0.083	-0.006	-0.153	0.091	0.396	0.248	0.416
	[0.37]	[-0.03]	[-0.55]	[0.79]	[1.28]	[1]	[1.21]
Stay-at-home requirements (c6)	-0.246	-0.162	-0.265	0.537	-0.270	0.014	-0.333
	[-1.43]	[-0.9]	[-1.31]	[3.89]***	[-1.3]	[0.07]	[-1.48]
Restrictions on the internal movement (c7)	0.151	0.139	0.149	0.097	0.149	0.093	0.157
	[1.56]	[1.05]	[1]	[1.14]	[1.07]	[0.73]	[1.03]
International travel controls (c8)	0.673	0.719	0.483	-0.517	0.847	0.966	0.772
	[3.91]***	[4.01]***	[2.36]**	[-2.71]***	[4.32]***	[3.42]***	[4.14]***
Lagged confirmed case	0.086	0.091	0.039	-0.201	0.065	-0.006	0.081
	[1.46]	[1.49]	[0.58]	[-3.72]***	[0.86]	[-0.1]	[1]
Intercept	4.536	4.353	3.245	4.385	2.075	1.415	2.146
	[9.37]***	[8.82]***	[5.73]***	[10.46]***	[4.29]***	[2.5]**	[4.47]***

This table presents coefficient estimates and t-statistics in square brackets of the following regression $Y_{t}=\beta_{0}+\Sigma_{i=1}^{8}\beta_{i}c_{i,t}+\gamma \;Confirmed\;Case_{t-1}+\epsilon_{t}$. The intraday transaction data of all stocks listed in HSX is obtained over January to September 2020 to calculate the market quality measures. The quoted spread is simply the difference between the bid and ask prices, and the effective bid-ask spread is defined for each trade as twice the absolute difference between the trade price and the mid-point of the contemporaneous bid and ask quotes. The realized spread is computed as twice the amount by which prices for customer buy orders exceed, or prices for customer sell orders fall short of, the estimated post-trade value of the asset. The quotation size is simply the size of bid and ask quote recorded during a trading day. The traded spread and its components, namely inventory plus adverse selection cost and order-processing cost, are estimated following Huang and Stoll (1997). To aggregate the market-wide level of market quality measures, a simple mean is first computed on a stock daily basis, except for the quoted spread and quotation size that are time-weighted average. The cross-sectional daily mean is then calculated to have a time series observation. The daily number of a newly confirmed case, stringency index, and containment and closure policies are obtained from the Coronavirus Resource Centre website of the John Hopkins University and the Oxford COVID-19 Government Response Tracker (OxCGRT) database, respectively. The Apple’s mobility indexes on driving and walking of users in Ha Noi, Vietnam’s capital city and Ho Chi Minh city, the country’s economic and business centre, are summed up to be the aggregate mobility index. Standard errors are adjusted for heteroskedasticity, and the regression also includes the day-of-the-week dummies.

***, **, and * represent the statistical significance at 1 per cent, 5 per cent, and 10 per cent levels, respectively.

In a nutshell, we document a net positive impact on the aggregate containment and closure policy’s market quality measured by the *stringency* index. When the individual policies are studied together, the market quality reacts positively to a higher degree of restrictions on gatherings and cancellation of public events, but negatively to a more restrictive *school closing* and *international travel controls*. This suggests that policymakers should contemplate such policies to avoid any negative impact on the stock market. Lastly, the number of the newly confirmed case does not yield any negative impact on market quality when controlled for government response in the containment and closure policies.

## 5. Concluding remarks

This study examines the effect of the Vietnamese government policies on containment and closure to the quality of the stock market during the Covid-19 over January-September 2020 period. Various measures for market quality are used including quoted spread, effective spread, realized spread, quotation size, and the traded spread and its two sub-components, namely inventory plus adverse-selection cost and order-processing cost, are used for different types of trading costs. In addition, different containment and closure policies are considered such as school closing, workplace closing, cancel public events, restrictions on gatherings, close public transport, stay-at-home requirements, restrictions on the internal movement and international travel controls. Our findings indicate market quality is positively affected by some of the government’s containment and closure policies during the Covid-19 pandemic as follows. First, the market quality appears to be strongly improved with workplace closing policies, restriction on gatherings, close public transports, and stay-at-home requirements. Second, international travel appears to provide no effect on market quality. Lastly, school closing negatively impacts the market quality. Our findings support the Vietnamese government’s current policy in relation to gradually opening the economy by allowing various groups of foreigners to come to Vietnam. This policy plays an important role to support the economy which has been savaged by the current pandemic since the beginning of 2020.

The outbreak of COVID-19 in late December 2019 in Vietnam and later ascended to global pandemic has resulted in two worldwide social and economic distresses. To January 2021, there were already 99 million confirmed cases, 2.5 million deaths around the world. Vietnam is one of the world’s best countries who have well-controlled and contained the transmission rate of the infectious virus. The success is mainly attributed to the government’s efforts in swift decision-making, effective public health messaging and aggressive contact tracing. We consider some policies implemented in Vietnam for other countries that have been still fighting hard to control the current Covid-19 pandemic. These policies, which are supported by empirical evidence from our analysis, are discussed in turn below.

First, the Vietnamese government had directed people not to go for large gathering, events and festivals. Even when the virus is under control, wearing a face mask in a public space is required at all times. People who refuse to wear face masks in public places are provided with strict penalties. The decision might have caused massive damage to multiple industries such as aviation, recreation, tourism and finance. The most restrictions had been applied between July and August 2020 following the second outbreak in Da Nang’s city in the central part of Vietnam. We consider that the outbreaks in Vietnam, mainly during the July-August 2020 period, highlight the role of vigilance and carefulness that the reduction in newly confirmed cases does not go in tandem with no virus transmission among society.

Second, policies on lockdown of the entire economy, which was between April 1 to April 15 2020, play an important role in containing the spread of this deadly virus. Different business sectors were affected distinctively during the COVID-19 pre-lockdown and during the lockdown in Vietnam. The financial sector is the hardest hit sector due to the possible occurrence of excessive bad loans and massive capital withdrawals. However, the lockdown enhances the confidence of investors by showing the government’s determination to control the pandemic.

Third, the Vietnamese government has been very successful with the so-called policy on contract tracing. A phone application called “Blue-zone” was developed, and its use is compulsory in Vietnam. Blue-zone has received more than 50 million downloads, more than half of Vietnam’s total population and thousands of suspected people of infection have been traced.

For future research, it is interesting to study how the market quality is affected by openness risk, policies in economic support, or government responses in different countries whose residents have different attitudes towards the policies against COVID-19. For example, while the number of death and confirmed cases support Vietnam as a leading country with strict and immediate responses to the COVID-19, many other countries face difficulties in imposing social distance-related policies.

## Supporting information

S1 Data (ZIP)
